# 2,4-Dinitrotoluene (DNT) Perturbs Yolk Absorption, Liver Development and Lipid Metabolism/Oxygen Transport Gene Expression in Zebrafish Embryos and Larvae

**DOI:** 10.3390/ijms20153632

**Published:** 2019-07-25

**Authors:** Jianglin Xiong, Hang Sha, Hualin Zhou, Lijuan Peng, Lingying Wu, Yinsheng Qiu, Rui Wang, Xianqin Hu

**Affiliations:** 1 Hubei Key Laboratory of Animal Nutrition and Feed Science, Wuhan Polytechnic University, Wuhan 430023, China; 2 Yangtze River Fisheries Research Institute, Chinese Academy of Fishery Sciences, Wuhan 430223, China; 3 Agricultural College, Xiangyang Vocational and Technical College, Xiangyang 441050, China; 4 School of Food Science and Engineering, Wuhan Polytechnic University, Wuhan 430023, China

**Keywords:** 2,4-dinitrotoluene, zebrafish (*Danio rerio*), embryos, liver, yolk

## Abstract

2,4-dinitrotoluene (2,4-DNT) is a common environmental pollutant, and was classified as a group 2B human carcinogenic compound by the International Agency for Research on Cancer. This study determined the toxic effects of 2,4-DNT exposure on zebrafish at the embryo-larvae stage, in terms of organ morphogenesis and the expression pattern of selected target genes related to lipid metabolism and oxygen transportation. The results showed that the 120-h post-fertilization LC_50_ of 2,4-DNT was 9.59 mg/L with a 95% confidence interval of 8.89–10.44 mg/L. The larvae treated with 2,4-DNT showed toxic symptoms including smaller body, less skin pigment production, yolk malabsorption, and disordered liver development. Further studies on the expression of genes related to lipid transport and metabolism, and respiration indicated that they were significantly affected by 2,4-DNT. It is concluded that 2,4-DNT exposure perturbed liver development and yolk absorption in early-life zebrafish, and disturbed the lipid metabolism /oxygen transport gene expression.

## 1. Introduction

2,4-Dinitrotoluene (2,4-DNT) belongs to the family of nitrobenzene compounds, and it is widely used in the production of polyurethane, dyes, pesticides and rubber, and is the main component in specific explosive mixtures [1,2]. Exposure to 2,4-DNT can induce cancer in laboratory animals and may result in cancer in humans, as 2,4-DNT was classified as a group 2B human carcinogenic compound (possibly carcinogenic to humans) by the International Agency for Research on Cancer of the World Health Organization [3], and was listed as a priority pollutant by the U.S. Environmental Protection Agency [4]. DNT-contaminated water may be generated during production, assembly and packing in commercial and military industries, and can then enter rivers, lakes, soil, and other water resources [5,6]. It was also documented to contaminate rivers and sediments and is widely distributed in surface waters in China, such as the Songhua River and Yangtze River [7,8]. 2,4-DNT is difficult to degrade, persists in water, and is toxic to aquatic and terrestrial organisms, and humans [4,9,10]. The toxic effects of 2,4-DNT in wildlife include methemoglobinemia, anemia, reticulocytosis, Heinz bodies, hepatocellular carcinoma, and degenerative lesions of the cerebellum [11]. Xu and Jing (2012) found that 2,4-DNT exposure decreased enzyme activity and energy reserves in the liver tissues of common carp, and negatively affected the growth process [10]. Moreover, Wint et al. (2006) studied gene expression profiles in 2,4-DNT-challenged adult fathead minnow using cDNA microarray, and 2,4-DNT was found to only affect a few genes due to limited genomic information in fathead minnow [12]. By contrast, the whole genomic information on zebrafish (*Danio rerio*) has been disclosed, and these fish are well suited as model organisms for acute and chronic toxicity assays in aquatic toxicity analyses [13].

2,4-DNT pollution and its toxicity are a concern worldwide. Although several studies have reported diseases and toxicity related to 2,4-DNT, to date, few studies have determined the toxic effects of 2,4-DNT on embryonic development. Therefore, the objective of the present study was to evaluate the toxic effects of 2,4-DNT on liver development and yolk absorption during the early life of zebrafish, including organ morphogenesis and the expression pattern of selected target genes involved in lipid metabolism and oxygen transportation.

## 2. Results

### 2.1. Mortality of Zebrafish Larvae from 24 to 120 h Post-Fertilization (hpf)

**Figure 1:**
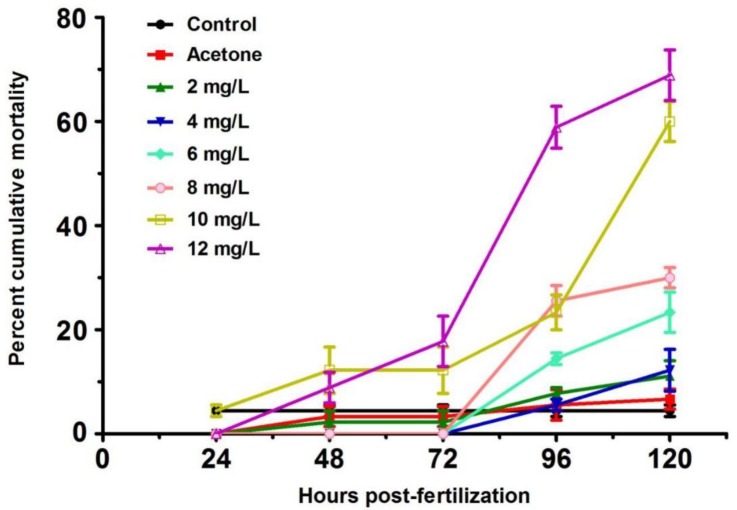
Cumulative mortality of zebrafish larvae exposed to 2,4-DNT-free (control) acetone-only (acetone) and 2, 4, 6, 8, 10, and 12 mg/L of 2,4-DNT.

Zebrafish larvae are sensitive to toxins at an early stage of development. As a group 2B human carcinogenic agent, 2,4-DNT was studied for its toxic effect on zebrafish larvae from 24 to 120 h post-fertilization (hpf). There was no difference in mortality between the control and vehicle group (acetone) during the experimental period (Figure 1). Mortality of larvae exposed to 2,4-DNT was low from 24 to 72 hpf. Notably, embryos exposed to 10 and 12 mg/L of 2,4-DNT significantly increased (*p* < 0.05) from 96 to 120 hpf, and reached 60% and 69%, respectively, at 120 hpf. From the experimental data acquired in this study, the relationship between mortality (y) and 2,4-DNT concentration (x) can be described by the following equation: y (%) = −0.0963 + 0.0627x (mg/L), R^2^ = 0.9109; *p* < 0.001. The 120-hpf LC_50_ of 2,4-DNT was 9.51 mg/L with a 95% confidence interval (CI) of 8.89–10.44 mg/L based on the linear regression analysis on larvae mortality and 2,4-DNT concentration. 

### 2.2. 2,4-DNT Induced Embryonic Malabsorption Syndrome at 3 Days Post-Fertilization (dpf)

**Figure 2:**
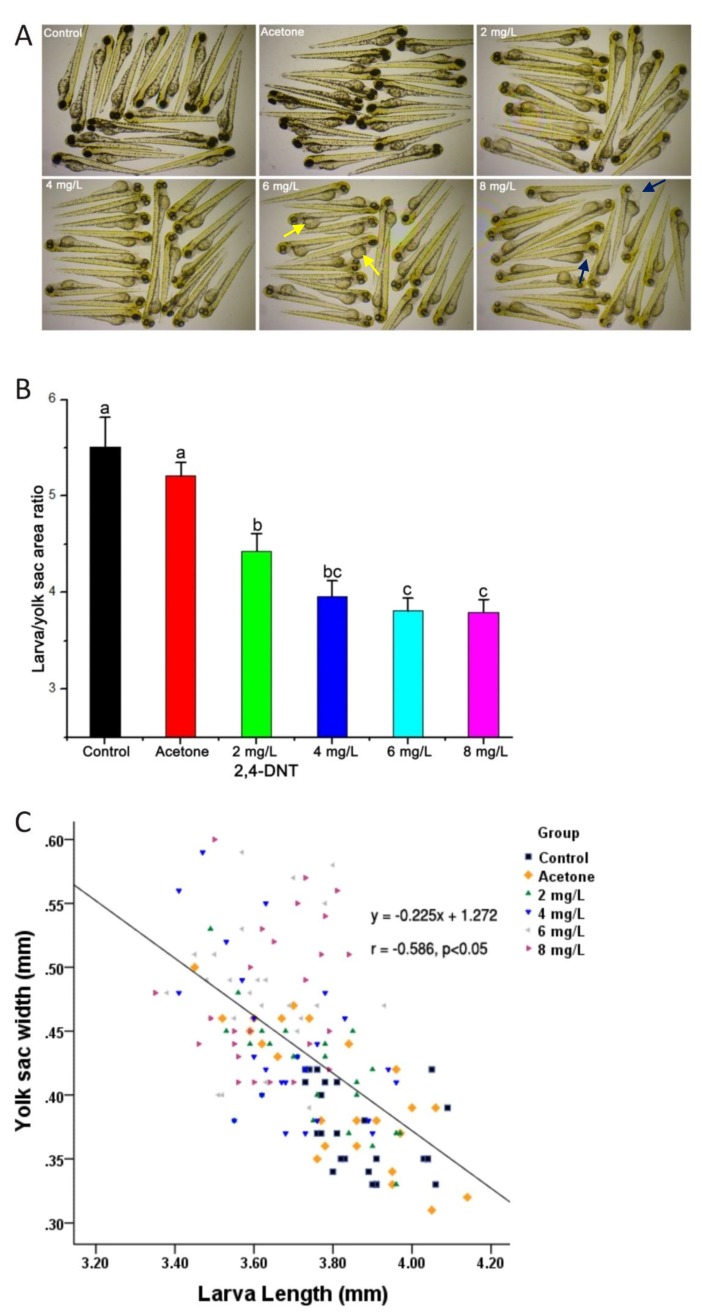
Morphogenesis of zebrafish larvae exposed to different concentrations of 2,4-Dinitrotoluene (2,4-DNT). **A**: The morphology of larvae exposed to 2,4-DNT from 2 hpf (hours post-fertilzation) to 3 dpf (days post-fertilization). The pictures were taken with the stereomicroscope in 3× magnification. The larvae show light skin, yolk sac accumulation and pericardial edema. The yellow and blue arrows indicate the yolk sac and pericardial cavity, respectively. **B**: Larvae/yolk sac areas in the control and 2,4-DNT-treated larvae. Bars that do not share a common lower-case letter are significantly different between the treatments (*p* < 0.05). **C**: Relationship between the width of the yolk sac and the total length of the larvae.

In the early stage of embryonic development, zebrafish mainly rely on the endogenous nutrients in yolk to complete morphogenesis. Compared with the control group, zebrafish larvae exposed to 2,4-DNT were smaller, consumed less yolk, and had lighter skin at 3 dpf (Figure 2A). There was obvious pericardial edema in the larvae challenged with 8 mg/L of 2,4-DNT. Yolk consumption was estimated by the ratio of larval body to yolk sac areas based on the left lateral view. Compared with the blank control and vehicle group, 2,4-DNT significantly decreased (*p* < 0.05) this ratio, with a minimum value of 3.80 ± 0.13 in the 8 mg/L treated group (Figure 2B). An inverse correlation (r = −0.586) was observed between total larvae length and the width of the yolk sac areas on the dorsal view (Figure 2C). Morphometric analyses demonstrated that exposure to 2,4-DNT resulted in a reduction (*p* < 0.05) in body length and yolk consumption, compared with the blank control and vehicle group.

### 2.3. 2,4-DNT Impaired Endotrophic Lipid Consumption in Zebrafish Larvae at 3 dpf

**Figure 3:**
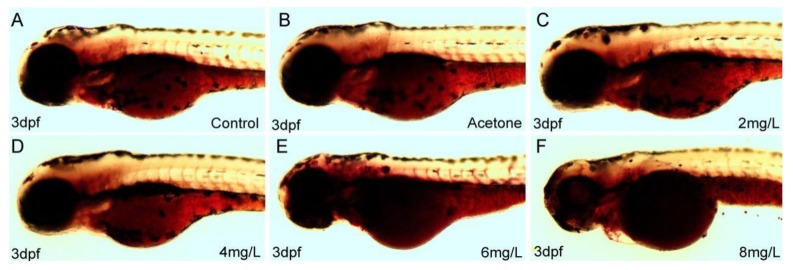
Oil red O (ORO) staining of representative larvae is shown in the left lateral view following different treatments at 3 dpf. **A**: Control larvae at 3 dpf; **B**: Vehicle control larvae treated with 0.05 mL/L acetone at 3 dpf; **C**: 2,4-DNT 2 mg/L-treated larvae at 3 dpf; **D**: 2,4-DNT 4 mg/L-treated larvae at 3 dpf; **E**: 2,4-DNT 6 mg/L-treated larvae at 3 dpf; **F**: 2,4-DNT 8 mg/L-treated larvae at 3 dpf. The pictures were taken with the stereomicroscope in 5× magnification

Oil Red O (ORO) is a neutral lipid dye, and used for staining zebrafish larvae in this study. Oil Red O staining zebrafish can exhibit lipid malabsorption in yolk. Whole-mount ORO staining of representative larvae showed staining in the anterior section on the left lateral view (Figure 3). 

**Figure 4:**
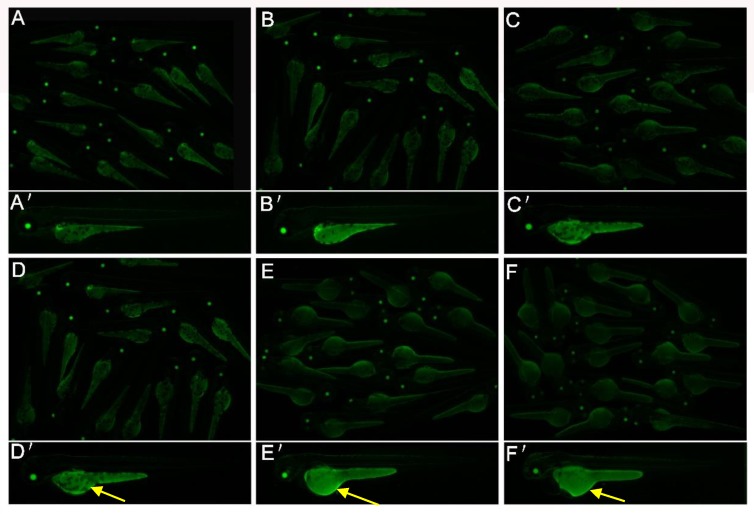
Yolk malabsorption in *Tg(apop:GFP)* larvae exposed to 2,4-DNT at 3 dpf. **A**: blank control; **B**: vehicle control (0.05 mL/L acetone); **C**: 2 mg/L 2,4-DNT; **D**: 4 mg/L 2,4-DNT; **E**: 6 mg/L 2,4-DNT; **F**: 8 mg/L 2,4-DNT. The yellow arrows indicate yolk sac accumulation. The pictures for A-F were taken with the stereomicroscope in 2.5× magnification, and A’-F’ were in 3.5× magnification.

Larvae exposed to 2,4-DNT exhibited a larger area and stronger ORO staining in the yolk sac compared with the blank control and vehicle group. In addition, there was evidence of a dose-dependent relationship between 2,4-DNT concentration and the degree of ORO staining. The 3-dpf *Tg(apop:GFP)* transgenic larvae (generated by microinjection in the Institute of Hydrobiology, Chinese Academy of Sciences) exposed to 2,4-DNT were analyzed in vivo using a green fluorescent protein (GFP) filter on a stereomicroscope (Figure 4). The transgenic larvae treated with 2–8 mg/L of 2,4-DNT consumed very little yolk, which was consistent with the results of whole-mount ORO staining of larvae. Thus, exposure to 2,4-DNT contributed to impaired lipid consumption in zebrafish larvae at 3 dpf. 

### 2.4. 2,4-DNT Inhibited Liver Organogenesis in 5 dpf Larvae

**Figure 5:**
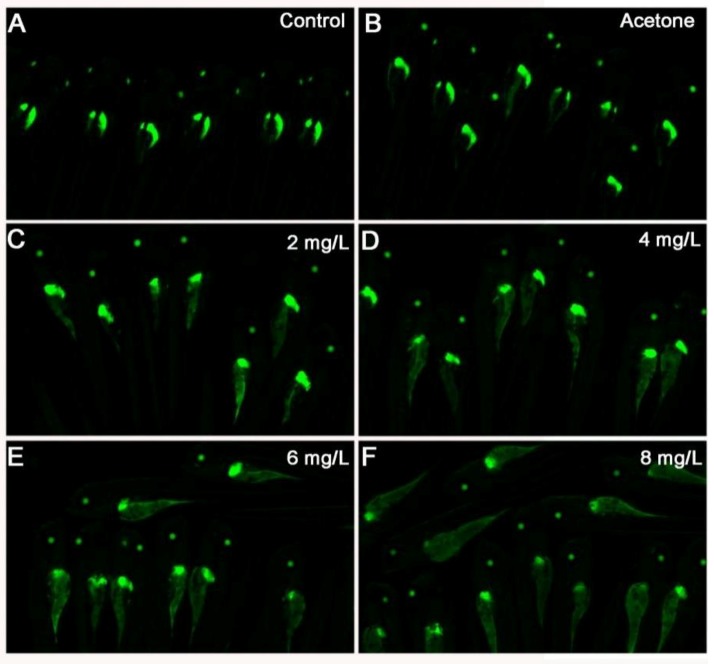

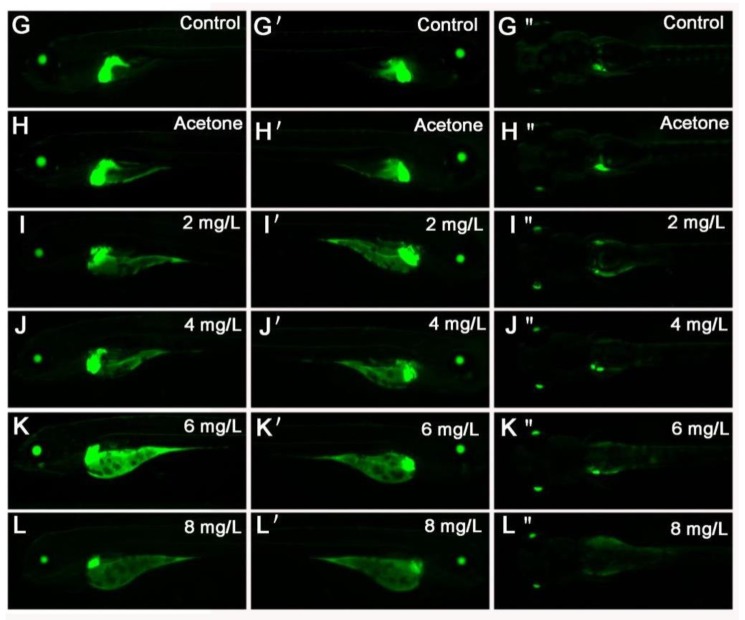
2,4-DNT inhibited the development of hepatic primordium in *Tg(apop:GFP)* larvae at 5 dpf. **A**: blank control; **B**: vehicle control (0.05 mL/L acetone); **C**: 2 mg/L 2,4-DNT; **D**: 4 mg/L 2,4-DNT; **E**: 6 mg/L 2,4-DNT; **F**: 8 mg/L 2,4-DNT; **G**–**L**: left view; **G’**–**L’**: right view; **G’’**–**L’’**: dorsal view. The pictures for A-F were taken with the stereomicroscope in 3.5× magnification, and G-L, G’–L’ and G’’–L’ were in 4.5× magnification.

*Tg(apop:GFP)* transgenic larvae can specifically express GFP in the liver and yolk sac. Analysis of GFP can intuitively report toxic effect of 2,4-DNT on liver development and yolk absorption. As shown in the blank control and vehicle control (Figure 5A,B), the bi-lobed boomerang-shaped liver in 5 dpf larvae consisted of a larger left lobe which crossed the midline under the anterior gut, and a smaller right lobe which extended ventrally towards the head of the pancreas. However, liver growth in 5 dpf larvae was stunted following exposure to 2,4-DNT, and the yolk sac was not consumed (Figure 5C–F). Larvae treated with 2 and 4 mg/L of 2,4-DNT exhibited an irregular shaped left lobe and the right lobe was smaller than that in the control (Figure 5C,D). Compared with the control group, larvae exposed to 6 and 8 mg/L of 2,4-DNT displayed a small left lobe and the right lobe had almost disappeared, these findings were more evident following exposure to 8 mg/L of 2,4-DNT (Figure 5L–L”). The results indicate that exposure to 2,4-DNT induced disordered liver development, which was closely associated with the concentration of 2,4-DNT.

### 2.5. Effects of 2,4-DNT on the Expression of Genes Related to Lipid Transport and Lipolysis

**Figure 6:**
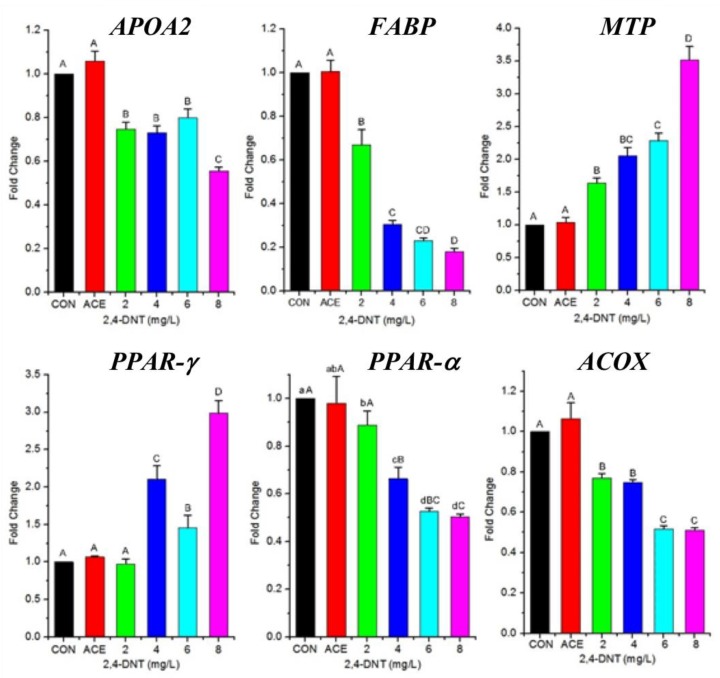
Relative mRNA expression levels of genes involved in lipid transport and metabolism in zebrafish exposed to 2,4-DNT at 5 dpf. Abbreviations: CON: control group; ACE: acetone group; APOA2: apolipoprotein a 2; FABP: fatty acid binding protein; MTP: microsomal triglyceride transfer protein; PPAR-*γ*: peroxisome proliferator-activated receptor γ; PPAR-*α*: peroxisome proliferator-activated receptor α; ACOX: acyl-CoA oxidase; unlike lower-case letters, superscripts for mean values are significantly different between the groups (*p* < 0.05). Bars that do not share a common capital letter are significantly different between the treatments (*p* < 0.01).

The previous trial showed 2,4-DNT impaired endotrophic lipid consumption. Thus, we tried to further study effects of 2,4-DNT on the expression of several genes in cDNA library reported by Wint, et al. (2006), involved in lipid metabolism. Exposure to 2,4-DNT affected the mRNA levels of genes related to lipid transport and lipolysis on day 5 (Figure 6). The main bioactive proteins including apolipoprotein a II (APOA2 coding, involving in lipid transport), fatty acid binding protein (FABP, lipid transport), microsomal triglyceride transfer protein (MTP, lipid transport), acyl-coenzyme A oxidase (ACOX, fatty acid β oxidation) and peroxisome proliferator-activated receptor α and γ (PPAR-α, lipid transport; PPAR-γ, oxidative decomposition of lipids) participate in lipid transport and metabolism. In the present study, APOA2 and FABP mRNA levels in larvae in the 2,4-DNT groups were significantly lower (*p* < 0.05) than that in the control group. The expression level of MTP was consistently upregulated when exposed to the higher level of 2,4-DNT (*p* < 0.05) when compared with the control group. The mRNA level of PPAR-γ was significantly (*p* < 0.05) increased in 2,4-DNT-treated larvae when compared to the control group, whereas PPAR-α and ACOX mRNA expression in 2,4-DNT-treated larvae were significantly (*p* < 0.05) downregulated compared with the control group. These results indicated that 2,4-DNT changed the mRNA levels of genes involved in lipid transport and lipolysis.

### 2.6. 2,4-DNT Up-Regulated Transcription of Genes Involved in Respiration

**Figure 7:**
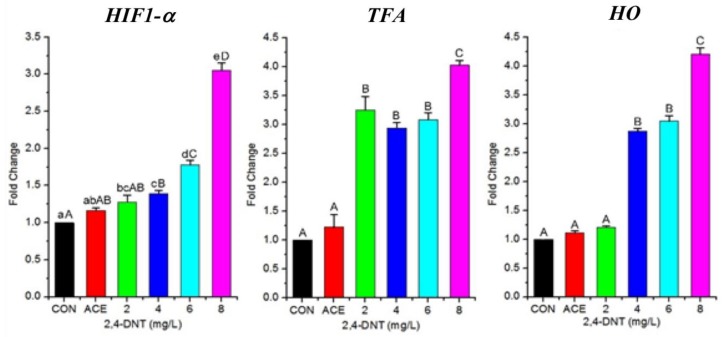
Relative mRNA expression levels of genes involved in respiration in zebrafish larvae exposed to 2,4-DNT at 5 dpf. Abbreviations: CON: control group; ACE: acetone group; HIF1-*α*: hypoxia inducible factor 1, alpha subunit; TFA: transferrin-a; HO: heme oxygenase; values are means ± SEM (*n* = 3). Bars that do not share a common lower-case letter are significantly different between the treatments (*p* < 0.05). Bars that do not share a common capital letter are significantly different between the treatments (*p* < 0.01).

During this study, the larvae treated with 2,4-DNT were found in surface water. This clinical manifestation indicated 2,4-DNT induced hypoxia in zebrafish. Thus, further trial was carried out to effects of 2,4-DNT on the expression of several genes in cDNA library reported by Wint, et al. (2006), involved in respiration. Transcription of the following three genes: hypoxia inducible factor 1-a (HIF1-a), transferrin-a (TFa) and heme oxygenase (Ho), related to respiration, was analyzed (Figure 7). The level of HIF1-a mRNA expression in the 2,4-DNT-treated groups was significantly (*p* < 0.05) higher than that in the control group. The mRNA expression level of TFa, involved in serum iron transport, was significantly increased following exposure to 2,4-DNT. In addition, Ho mRNA expression was upregulated (*p* < 0.05) in 2,4-DNT exposed larvae when compared with controls. Thus, on the basis of mRNA expression level, 2,4-DNT upregulated genes involved in respiration.

## 3. Discussion

In recent years, nitrotoluene chemicals have been shown to affect humans and other organisms, and have caused worldwide concern due to their widespread use and unknown risks. Of these chemicals, 2,4-DNT is a suspected carcinogen [4], which is present in environmental media including air, surface water, ground water, soil, and sediment [10,14], and is harmful to aquatic animals, humans, and other organisms. Serious lesions including fibroma, hepatocellular carcinoma, adenoma, and mammary tumors have been found in experimental rats chronically exposed to 2,4-DNT [11]. However, little information is available on 2,4-DNT-induced morphological and molecular toxicology in early-life fish. In recent years, zebrafish models have been widely used to study human disease, toxicology, and in drug screening assays [13,15] on the basis of their fecundity, uniformity, rapid external development and low cost. Newborn zebrafish larvae are sensitive to hazardous substances, and are suitable experimental toxicity models. The present study showed that 2,4-DNT induced a toxic response syndrome, which included yolk-sac malabsorption, growth retardation, morphological swimming abnormalities, and embryo lethality. Exposure to 2,4-DNT has been shown to result in acute, subacute, subchronic, and chronic toxicity in mammals, birds, amphibians, and reptiles at different concentrations, and caused decreased body weight, liver lesions, and death [11]. The LC50 of 2,4-DNT in zebrafish larvae was 9.59 mg/L at 120 hpf, lower than that for juvenile carp at 96 h (20.03 mg/L, [12]) and for bull-frog tadpoles at 96 h (40.29 mg/L, [16]). The different LC_50_ values of 2,4-DNT in organisms may be due to species, growth phase and challenge time. 2,4-DNT was found in wastewaters from TNT manufacturing facilities at levels of 0.04–48.6 mg/L [17], and thus should be strictly supervised and controlled. 

ORO staining of neutral lipids allows visualization of endogenous lipid consumption by fish at the embryonic and larval stages to provide an overall picture of neutral lipid localization in whole fixed fish [18]. The present study showed that exposure to 2,4-DNT resulted in strong ORO staining of the yolk sac with little staining of other structures. Thus, we speculate that 2,4-DNT exposure may impair normal lipid transport and metabolism. In addition, stunted growth observed in 2,4-DNT-exposed embryos and larvae was probably due to their inability to deliver yolk sac nutrients to the circulatory system as zebrafish embryos and larvae rely entirely on the yolk sac to obtain amino acids and lipids for growth and survival [19]. The exact mechanism of embryonic malabsorption induced by 2,4-DNT requires further investigation.

The liver is an essential organ in the body, and plays a vital role in metabolism and detoxification. DNT was reported to bio-concentrate and bio-accumulate in fish (common carp) liver [20]. Previous studies indicated that 2,4-DNT increased liver weight due to increased phospholipids in rats and fathead minnow [12,21]. Up until now, no information has been available on 2,4-DNT-induced morphogenesis in fish liver. In this study, we were able to visualize liver morphogenesis in transgenic larvae in vivo exposed to 2,4-DNT at 5 dpf. We found that exposure to high dose 2,4-DNT (6 and 8 mg/L) can result in liver dysplasia, and may directly impair its biological functions. 

Compared with common biometric indices such as survival and appearance, gene expression profiling is more sensitive to toxicants [12] and may be used as an indicator of adverse phenotypic effects in identifying environmental pollutants. On the basis of the fathead minnow (*Pimephales promelas*) cDNA library reported by Wintz et al. (2006) [12], we detected the expression of several genes involved in lipid metabolism and oxygen transport, which may be related to yolk lipid absorption disorder and difficult respiration found in our study. 

PPARα is known to regulate liver lipid metabolism by transcriptionally activating lipid metabolism-associated genes (i.e., fatty acid oxidation genes) [22]. Our study showed that 2,4-DNT significantly decreased PPAR-α transcription in early-life zebrafish. Consistent with our findings, previous studies demonstrated that 2,4-DNT perturbed PPAR-α-dependent lipid metabolism signaling, and impaired lipid metabolism and energy budgets in fathead minnow and mice [12,23]. Similar responses were also observed following exposure to multiple nitrotoluenes including TNT, 2,4-DNT, and 2,6-DNT in northern bobwhite, rats, mice, and Daphnia magna [24]. Given that PPAR-α is a primary nuclear transcriptional regulator of lipid metabolism, the depression of *PPAR-α* mRNA may be responsible for the disordered lipid transport and metabolism seen in 2,4-DNT-treated larvae. 

PPAR-γ, a member of the PPAR family, was also reported to regulate milk fat metabolism in bovine mammary epithelial cells [25,26] and modulate lipid accumulation by regulating the transcription of the adipose differentiation-related protein (ADRP) gene in goat mammary epithelial cells [27]. The present study also showed that PPAR-γ transcription tended to increase in 2,4-DNT-challenged larvae, which may be a compensatory response to energy deficits in the absence of PPAR-α as activated PPAR-γ promotes oxidative metabolism of lipids [28]. In accordance with this study, the expression of *PPAR-γ* increased in *PPAR*-α(−/−) mice treated with 2,4-DNT [23].

Besides PPARs, other important genes including *APOA2, FABP, ACOX,* and *MTP* are also involved in lipid transport and metabolism. APOA2, the second most abundant constituent of HDL, plays an important role in lipid transport and metabolism including visceral fat accumulation and metabolism of triglyceride-rich lipoproteins, and has been related to obesity [29]. In addition, FABPs can bind long-chain fatty acids and other ligands, and are recognized to participate in fatty acid metabolism and transport. Aberrant FABP expression may be regarded as a potential mediator of tumorigenesis [30]. Moreover, acyl-CoA oxidase plays a key role in fatty acid *β*-oxidation. Our study showed that 2,4-DNT downregulated the expression of *APOA2, FABP,* and *ACOX* genes suggesting that this toxicant interferes with lipid transport and metabolism. Besides the effects of 2,4-DNT on the expression of these genes in fathead minnow, it also induced yellow liver with a higher fat content [12]. 

Interestingly, we observed significant upregulation of MTP, which was related to very low-density lipoprotein (VLDL) assembly and secretion. Activated PPARγ can inhibit the production of inflammatory factors such as TNF-α, IL-1, IL-2, and IL-6 [28]. Of these factors, IL-1 and IL-6 significantly decreased MTP mRNA levels in HepG2 cells [31]. Thus, these previous studies could explain why MTP expression had an indirect positive correlation with PPARγ in this study.

In addition, zebrafish developed an oxygen deficit when exposed to 2,4-DNT, therefore, we presume that some genes may be differently expressed in response to disordered oxygen transport. The following three genes *HIF* 1-α, *TFA,* and *HO* have been reported to be associated with oxygen transport [32,33,34,35]. Oxygen deficiency due to 2,4-DNT exposure may regulate the expression of these three genes. This study showed that the *HIF 1-*α gene was significantly downregulated when exposed to 2,4-DNT, and 2,4-DNT may result in hypoxia in larvae. A previous study indicated that HIF 1-α was a main regulator of the cellular response to hypoxic stress, and its transcriptional activity could be induced by hypoxic conditions [35]. The *TFA* gene was reported to be induced by hypoxia in mammals, and its transcription can be activated by hypoxia-inducible factor [33]. The *TFA* gene, which mediates cellular iron uptake, was significantly upregulated in our study, which showed that 2,4-DNT is a hypoxia-inducible factor and causes hypoxia. In addition, a significant increase in the expression level of *HO* mRNA was observed in zebrafish larvae exposed to 2,4-DNT. Heme oxygenase-1 can cleave the heme ring to release iron ions, and *HO* was a target gene for HIF-1 induced by hypoxia in animal tissues and cell cultures [32]. 2,4-DNT was reported to cause methemoglobinemia by oxidizing hemoglobin ferrous iron to its ferric state [36], and directly impaired the oxygen-carrying capabilities of hemoglobin. Thus, the ability of blood to transport oxygen was likely reduced, and subsequently resulted in functional hypoxia in 2,4-DNT-exposed zebrafish larvae. The expression of these three genes was induced by 2,4-DNT in zebrafish larvae, and may result in functional hypoxia.

By analyzing lipid metabolism/oxygen transport gene expression, we found significant changes in gene expression following exposure to 2 mg/L of 2,4-DNT, whereas the toxicant at this concentration did not significantly affect fish survival. Thus, gene expression may be more sensitive to toxicants than survival rate. However, further studies on these issues are necessary, such as the effect of 2,4-DNT at lower concentrations on gene expression and the association of gene expression with phenotypes.

## 4. Materials and Methods

### 4.1. Fish Breeding

The experiments were conducted at the Aquatic Biology Laboratory of Wuhan Polytechnic University, China, and were in accordance with the guidelines established by the Animal Care and Use Committee of Hubei Province. All experiments carried out were approved by the Ethics Committee of the Wuhan Polytechnic University in Hubei Province, China (number WPU-F20150701, approval date 1 July, 2015)

The wild-type AB line zebrafish (*Danio rerio*) used were obtained from China Zebrafish Resource Center, and the *Tg*(*apop:GFP*) transgenic line zebrafish were generated by microinjection in the Institute of Hydrobiology, Chinese Academy of Sciences using the methods described by Wang (2011) [37]. The *Tg*(*apop:GFP*) transgenic line zebrafish express green fluorescence protein to track yolk absorption and liver development. The fish were housed in a recirculating aquaculture system using aerated tap water, with a photoperiod of 14 h light:10 h dark at 27 ± 1 °C. The embryos were collected following natural fertilization of adult zebrafish (2 ♂: 1 ♀ in the spawning box) and staged according to the morphological criteria described by Kimmel (1995) [38].

### 4.2. Preparation of Chemicals and Solutions for Exposure Treatments

2,4-DNT (101397; ≥97% pure; Sigma-Aldrich, St. Louis, MO, USA) was dissolved in acetone (analytical grade; Sinopharm Chemical Reagent Co., Ltd., Shanghai, China) to prepare a 200 mg/mL 2,4-DNT stock solution. Acetone was diluted to obtain a 10 mL/L stock solution. Both stock solutions were stored at 4 °C, away from light.

### 4.3. Exposure Procedures

The exposure experiment consisted of the following six treatments: 2, 4, 6, 8, 10, and 12 mg/L of 2,4-DNT [similar to the 2,4-DNT levels described by Wint et al. (2006)], respectively, which were prepared by spiking the 2,4-DNT stock solution into aerated tap water. The final concentration of acetone in all exposure treatments was adjusted to 0.05 mL/L using the acetone stock solution. Acetone was found to have no discernable effect on embryo development in our preliminary study. In addition, a control group without 2,4-DNT and acetone, and an acetone group with 0.05 mL/L of acetone were also prepared. 

Within 2 h after spawning, the embryos were randomly assigned to 100 mL glass beakers containing the experimental solutions and placed in a dark incubator at 28.5 °C. At 6 hpf, developing embryos at the shield stage were selected and transferred to plastic 6-well plates with 20 embryos and 10 mL of experimental solution in each well. Each treatment included 20 embryos with four replicates of each treatment, three replicates for the wild-type line and one for the transgenic line, respectively. The embryos were reared in the incubator at 28.5 °C, away from light and checked every 12 h. The solutions were completely replaced twice each day, and fish mortality was recorded at the same time. 

### 4.4. Larvae Monitoring and Morphometric Analyses

Phenotypes were examined with an Olympus SZ61 stereomicroscope (Olympus Corporation, Tokyo, Japan). The 3 dpf and 5 dpf larvae were firstly anesthetized with 200 mg/L of tricaine (E10521; ≥98% pure; Sigma-Aldrich, St. Louis, MO, USA), and then left and vertical images were obtained using a CoolSNAP K4 camera (Acal BFi Limited Company, Uppsala, Sweden) attached to the stereomicroscope with the same magnification. Larval morphological features including total body length (from the anterior-most part of the snout to the posterior-most point of the tail), yolk sac area and body surface area (without yolk sac) were analyzed using Metamorph software (Universal Imaging Corp., Bedford Hills, NY, USA) based on the left lateral view. Yolk consumption was calculated by the ratio of larval body to yolk sac areas in the left lateral image. GFP fluorescent patterns of the transgenic larvae were analyzed using a Leica MZ16FA stereomicroscope (Leica Camera AG, Wetzlar, Germany) with a GFP filter.

### 4.5. ORO Staining

The three dpf larvae were fixed overnight in 4% paraformaldehyde at 4 °C, and then stained using the methods described by Schlombs (2003) [39]. Briefly, the larvae were progressively washed twice for 5 min each time in each of the following: 20, 40 and 60% (*v*/*v*) 2-propanol in PBS solution, and then stained for 2.5 h in freshly filtered 0.3% ORO (1320-06-5; Amresco Commercial Finance, LLC, Boise, ID, USA) as a neutral lipids dye. The stained larvae were then rinsed twice for 5 min each time with the following successive dilutions of 2-propanol in PBS solution: 20, 40 and 60% (*v*/*v*). The larvae were then placed in PBS solution, and left-sided images were obtained using the stereomicroscope.

### 4.6. Isolation of Total Larval RNA and Quantitative RT-PCR

Total RNA was isolated from 30 5-dpf larvae in each treatment using TRIzol Reagent (Invitrogen™, Thermo Fisher Scientific, Wilmington, DE, USA), and was then quantified using the NanoDrop ND-2000 (Thermo Fisher Scientific, Wilmington, DE, USA). The integrity of RNA in each sample was assessed using 1% agarose gel electrophoresis, which showed clearly visible 28S, 18S and 5S ribosomal RNA bands. Ribosomal RNA (1 μg) was reverse transcribed into cDNA using a PrimeScript**^™^** First Strand cDNA Synthesis Kit (Takara Bio Inc., Kusatsu, Shiga, Japan) when the 28 S:18 S ribosomal RNA ratio ≥ 1.8, and the cDNA was used as a template for quantitative PCR (q-PCR).

Key genes involved in lipid metabolism and hypoxic stress were chosen to determine their expression. Oligonucleotide PCR primers were designed according to the zebrafish sequences in the GeneBank database and are listed in Table 1. The q-PCR reactions were carried out with the FastStart Universal SYBR Green Master (Hoffmann-La Roche Ltd., Basel, Switzerland) on the Applied Biosystems 7500 Real-Time PCR System (Applied Biosystems, Inc., Foster City, CA, USA).

The 10 μL amplification system included 5 μL of SYBR Premix Ex Taq, 0.2 μL of 10 μmol/L forward primer, 0.2 μL of 10 μmol/L reverse primer, 0.2 μL of ROX reference Dye II, 3.4 μL of nuclease-free water and 1 μL of cDNA template. The q-PCR procedure was conducted as follows: 95 °C for 10 min, 40 cycles of 95 °C for 15 s, 60 °C for 30 s and 72 °C for 30 s, and simultaneous detection of fluorescence signals. The specificity of the amplification products was confirmed by sequencing at Shanghai Majorbio Bio-pharm Technology Co., Ltd. (Shanghai, China). PCR was conducted in triplicate for each sample to obtain the mean value of gene expression.

### 4.7. Statistics

All results are expressed as mean ± standard deviation (SD). The data were analyzed by one-way analysis of variance (ANOVA) using SPSS version 19.0 (SPSS Inc., Chicago, IL, USA). Duncan’s multiple range tests were carried out to determine significant differences between treatment means. The normality and constant variance of data were confirmed by Levene’s test. If the data did not exhibit homogeneous variance, the data were log-transformed to meet the necessary assumptions for ANOVA. All statements of statistical significance were set at *p* < 0.05, and trends were based on a probability of 0.05 < *p* ≤ 0.10. 

## 5. Conclusions

The results of this study demonstrated that 2,4-DNT exposure perturbs liver development and yolk absorption in early-life zebrafish with the following pathological features: disruption of morphogenesis, embryonic malabsorption syndrome, and stunted liver organogenesis. Additional findings indicated that 2,4-DNT exposure disturbed the PPAR-α-dependent lipid metabolism pathway and caused hypoxia by increasing the transcripts of hypoxia-inducible genes. Early-life zebrafish are model organisms sensitive to 2,4-DNT, and can be used to evaluate environmental 2,4-DNT pollution. 

## Figures and Tables

**Table 1:** Primers used in all experiments.

Primer Name	Sequence (5′–3′)
ZPPARGF	CGCAGGCTGAGAAGGAGAAGC
ZPPARGR	CATGTATCTGCAGTTGATCATC
ZPPARAF	CATCACCAGAGAGTTTCTGAAG
ZPPARAR	GCGGCGTTCACACTTATCGTAC
ZAOXF	AAGGACATCGAGCGAATGATG
ZAOXR	ACTATAAAAGAGTGGAGGCCG
APOF	ATGAAGCTGACATTCGCTCTC
APOR	TAGTGCTGGCTCAACTGCAG
ZFABPF	ATGGCCTTCAGCGGGACGTGG
ZFABPR	TGAGCTTCTTGCCGTCCATAG
ZMTPF	ATGAACATTTACGGTCAGAGC
ZMTPR	CACCACATTGATAGGATCTCC
ZHIF1AF	GTCAGCAAGAGCATGGGCCTC
ZHIF1AR	GAAGAACCTTCCACGTCGCAG
ZHOF	GCGGCAGAGAACACTGGCAGT
ZHOR	CTGCACTGCTGGGTGGTCTGC
ZTFAF	GAAGGTCCTGCTCATCTCTTTG
ZTFAR	CAGATAATTATTTAGTCCACCAG
ZHBAF	GAGTCTCTCTGCCAAAGACAAAG
ZHBAR	CGATTTTGCTGACAGCCTCAGC
ZACTINBF	CATGGATGAGGAAATCGCTGC
ZACTINBR	GTTAGTCACAATACCGTGCTC
